# Honokiol and Its Emerging Role in Breast Cancer Therapy

**DOI:** 10.3390/cancers18121989

**Published:** 2026-06-18

**Authors:** Santosh Kumar Singh, Manasvi Kondamudi, Avinash Ittuveetil, Melad N. Dababneh, Brian M. Rivers, Rajesh Singh

**Affiliations:** 1 Department of Microbiology, Biochemistry, and Immunology, Morehouse School of Medicine, Atlanta, GA 30310, USArsingh@msm.edu (R.S.); 2 South Forsyth High School, Cumming, GA 30041, USA; 3 Department of Chemistry, Georgia State University, Atlanta, GA 30302, USA; 4 Department of Pathology, University of Alabama at Birmingham, Birmingham, AL 35294, USA; mdababneh@uabmc.edu; 5 Cancer Health Equity Institute, Morehouse School of Medicine, Atlanta, GA 30310, USA; brivers@msm.edu

**Keywords:** honokiol, MDR, chemotherapy, endocrine therapy, immunotherapy

## Abstract

Honokiol (HNK), a bioactive compound found in *Magnolia* species, is a promising, multifunctional agent with therapeutic effects on breast cancer (BrCa). Preclinical evidence, including *in vitro* and *in vivo* studies, suggests that HNK inhibits essential oncogenic pathways and reduces oxidative stress, inflammation, metabolic reprogramming, and cancer stemness. HNK demonstrates synergistic activity with chemotherapy, endocrine therapy, targeted therapy, and immune checkpoint inhibitors, increasing sensitivity to treatment across models of ER+, PR+, and HER2+ BrCas, as well as triple-negative breast cancers (TNBC). Nanotechnological delivery systems enhance the solubility, bioavailability, and intratumoral accumulation of HNK, increasing its translational capacity. Although clinical data remain very limited, current evidence in humans is insufficient to draw definitive conclusions regarding the safety and efficacy of HNK. This review summarizes mechanistic, preclinical, and emerging clinical data, highlights challenges in formulation and pharmacokinetics, and anticipates future trends in incorporating HNK into multimodal therapy for BrCa.

## Simple Summary

This review examines honokiol (HNK), a natural compound derived from the Magnolia plant, as a potential therapeutic agent for breast cancer (BrCa). Preclinical studies demonstrate that HNK modulates key biological processes, including oxidative stress, inflammation, cell cycle progression, apoptosis, and metastasis. HNK has demonstrated efficacy across multiple BrCa subtypes and enhances responses to chemotherapy, endocrine therapy, targeted therapy, and immunotherapy, while also mitigating drug resistance. Recent advances in nanotechnology have improved the solubility and delivery of HNK. Nevertheless, clinical evidence remains limited, with only a small number of studies involving human subjects. In summary, HNK may serve as a multi-target adjunct therapy, but additional clinical trials are required to establish its safety and therapeutic efficacy in patients.

## 1. Introduction

Breast cancer (BrCa), a heterogeneous disease, remains the most frequently diagnosed malignancy among women worldwide and is a leading cause of cancer-related death. This disease is driven by dysregulated proliferation, metastatic potential, and complex tumor–microenvironment interactions [1]. Recent developments in surgery, chemotherapy, endocrine therapy, targeted therapy, and immunotherapy have improved outcomes, but recurrence and treatment resistance remain clinical challenges [2,3]. These limitations have increased interest in alternative strategies that enhance efficacy while reducing toxicity. Honokiol (HNK), a natural biphenolic compound derived from the bark of *Magnolia grandiflora*, has gained attention for its capacity to modulate various oncogenic pathways in cancers, including BrCa [4,5,6]. Standard treatments are often constrained by subtype-specific resistance and systemic side effects, highlighting the need for multi-target agents such as HNK.

Preclinical studies indicate that HNK interacts synergistically with standard treatments across the various BrCa subtypes [7,8]. HNK enhances the effects of agents such as paclitaxel (PTX) and doxorubicin (DOX) while sensitizing tumor cells to HER2- and estrogen receptor (ER)-directed therapies [9,10]. As a naturally derived compound, HNK demonstrates, in experimental systems, broad pathway inhibition with relatively low toxicity [4]. These properties suggest a role for HNK in overcoming intrinsic and acquired resistance to therapy [10].

Clinical evidence for HNK in the treatment of BrCa remains limited, with most data derived from preclinical models and early translational studies [2,4,5]. Advances in nanotechnology-based delivery systems have improved HNK stability and anti-tumor activity in preclinical settings [11,12,13]. These developments support the rationale for further clinical assessment of HNK.

This review summarizes preclinical and early clinical evidence on HNK’s effects on BrCa molecular processes and their potential treatment implications. Major databases, including NCBI-PubMed, Scopus, and Web of Science, were searched using keywords (e.g., “honokiol,” “breast cancer,” “endocrine resistance,” “chemotherapy,” “immunotherapy,” “targeted therapy”, and “nano therapy”, and related combinations), with results sorted by relevance, mainly focused on the past 10 years, with a few exceptions upon limitations of the current data. Clinical studies were identified by searching ClinicalTrials.gov and PubMed using the keywords “honokiol and breast cancer” and “honokiol in cancer.” Inclusion criteria prioritized peer-reviewed studies relevant to HNK, BrCa biology, and associated molecular mechanisms. Non-relevant or non-scientific sources were excluded, including non-peer-reviewed abstracts, except in cases where the full-text article was unavailable. We further highlight the interactions between HNK and standard therapies, including chemotherapy, endocrine therapy, targeted agents, and immune checkpoint inhibitors, as well as its capacity to address drug resistance across various BrCa subtypes.

## 2. Honokiol and Its Anticancer Mechanisms

HNK exhibits broad-spectrum anticancer activity against various subtypes of BrCa [14,15]. HNK exerts context-dependent effects on oxidative stress in BrCa, including TNBC models. HNK increases intracellular reactive oxygen species (ROS) levels in cancer cells, coinciding with a time-dependent loss of mitochondrial membrane potential (ΔΨm), indicating that ROS production is closely linked to mitochondrial damage [16]. Moreover, HNK, at low concentrations, displays cytoprotective and anti-inflammatory activity by suppressing pro-survival and inflammatory signaling pathways; in contrast, at high concentrations, it promotes a pro-oxidant state, elevating intracellular ROS beyond antioxidant capacity, and thereby inducing mitochondrial dysfunction and activating apoptotic pathways [16]. Consistent with a biphasic response, HNK exhibits dose-dependent activity in BrCa [10,17,18]. In ER+ (MCF-7) cells, HNK reduces the level of oxidative stress and inhibits pro-inflammatory signaling pathways that contribute to tumor development [4]. Mechanistically, HNK causes G_0_/G_1_ cell cycle arrest by downregulating cyclin D1 and CDK4, as well as promoting intrinsic apoptosis-like pathways marked by increases in caspase-3 and caspase-9 activities [4,19]. It further alters mitochondrial balance in favor of apoptosis by modulating the Bcl-2 family, reducing the anti-apoptotic Bcl-2 and Bcl-xL, and increasing the pro-apoptotic Bax [4]. The combination of these molecular events and their capacity to inhibit ROS-mediated DNA damage indicates that HNK exhibits antioxidant and pro-apoptotic activities in hormone-responsive BrCa cells.

In addition to ER-positive cancers, HNK demonstrates activity in more aggressive BrCa disease phenotypes. HNK suppresses the phosphorylation of STAT3 in MDA-MB-231 TNBC cells, leading to reduced invasion, migration, and EMT-associated markers, such as vimentin [20]. HNK downregulates signaling in HER2+ (SK-BR-3) cells, resulting in lower cell viability and higher apoptotic vulnerability, which relates to its relevance in HER2-induced oncogenic signaling [21]. Furthermore, HNK was found to activate the LKB1–AMPK axis and induce miR-34a expression in MCF7, SKBR3, and SUM149 cells, thereby inhibiting EMT, stemness, and oncogenic leptin signaling in an LKB1-dependent manner [22]. Moreover, HNK has been shown to effectively inhibit the hedgehog (Hh) pathway in TNBC models. In BrCa, the sonic hedgehog (SHH) ligand, one of three ligands along with Indian hedgehog (IHH) and Desert hedgehog (DHH), is often abnormally activated. This activation significantly increases cell proliferation and survival [23,24,25,26]. SHH plays a crucial role in regulating cell differentiation and tissue formation [27]. Remarkably, HNK reduces the survival of TNBC cells, specifically MDA-MB-231 and MDA-MB-468, in a dose-dependent manner, with IC50 values ranging from 5 to 100 μM [4,19,26]. This evidence demonstrates that HNK can induce apoptosis by suppressing essential components of the Hh pathway, including SHH [28], Gli1, and Ptch1 [26], as well as downregulating NF-κB [12,28], a key transcription factor that promotes tumor growth through hedgehog signaling [26]. This positions HNK as a promising candidate for targeted BrCa therapies.

Although these findings indicate significant potential, the anticancer effects of HNK arise from the simultaneous modulation of multiple signaling pathways rather than a single molecular target. This broad-spectrum activity, while advantageous in suppressing interconnected oncogenic networks, shows a relative lack of target specificity compared with conventional targeted therapies. Such pleiotropic effects complicate the identification of HNK’s primary mechanism of action and create challenges in translating preclinical efficacy into predictable clinical outcomes. Nevertheless, HNK’s capacity to regulate pathways involved in proliferation, survival, inflammation, and metastasis may offer therapeutic benefits in heterogeneous and treatment-resistant BrCa, where compensatory signaling frequently limits the efficacy of single-target agents. Thus, HNK’s multitarget nature represents both a potential advantage and a limitation in its therapeutic development.

**Figure 1:**
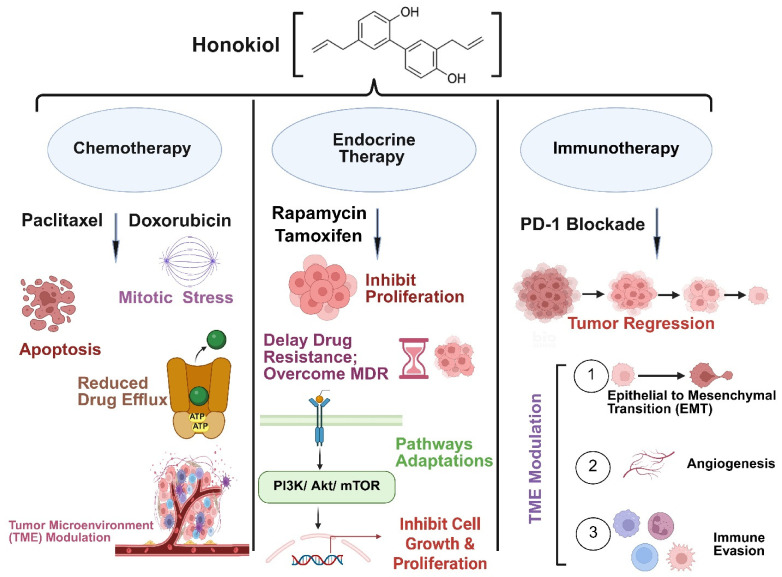
Mechanistic overview of honokiol (HNK) as a multimodal therapeutic modulator in BrCa. The figure depicts the integrative role of HNK across chemotherapy, endocrine therapy, and immunotherapy for BrCa, intended to provide a general framework encompassing multiple combinatorial strategies, rather than detailing pathway- or BrCa subtype-specific mechanisms. The detailed mechanistic distinctions and subtype-specific effects are thoroughly described in the main text. In the chemotherapy context (**left panel**), HNK enhances the efficacy of agents such as paclitaxel and doxorubicin by promoting mitotic stress-induced apoptosis, which involves activation of intrinsic apoptotic pathways and mitochondrial dysfunction. HNK also inhibits ATP-dependent drug efflux transporters, including ABC transporters, thereby increasing intracellular drug accumulation and overcoming multidrug resistance (MDR). Additionally, HNK modulates the tumor microenvironment (TME) by reducing inflammatory signaling and stromal support, further sensitizing tumor cells to cytotoxic agents. In the endocrine therapy setting (**middle panel**), HNK augments the effects of tamoxifen and mTOR inhibitors, such as rapamycin, by targeting critical survival pathways, including the PI3K/AKT/mTOR signaling pathway. This results in suppression of protein synthesis and cellular proliferation. HNK also delays the development of endocrine resistance and overcomes MDR by downregulating estrogen receptor (ER)-dependent and adaptive signaling networks. These mechanisms are particularly significant in ER-positive and hormone-resistant BrCa models, where pathway reprogramming facilitates therapeutic efficacy. In the immunotherapy context (**right panel**), HNK improves the response to immune checkpoint blockade, such as PD1 inhibitors, by promoting tumor regression and modulating immune surveillance. Mechanistically, HNK suppresses epithelial–mesenchymal transition (EMT), inhibits angiogenesis, and reduces immune evasion, thereby remodeling the TME to support anti-tumor immunity. These immunomodulatory effects are especially relevant in aggressive subtypes, including TNBC, where immune-based therapies are increasingly utilized. Collectively, these mechanisms establish HNK as a pleiotropic agent that enhances therapeutic response by coordinating the regulation of cell cycle progression, apoptosis, signaling pathways, drug resistance, and tumor–host interactions.

Evidence from experimental animals also supports the therapeutic promise of HNK. The 4T1 murine BrCa model demonstrates that HNK strongly inhibits tumor growth [29] and reduces the production of inflammatory cytokines, including TNF-α and IL-6 [30,31], supporting the concept that HNK regulates both oncogenic and inflammatory networks in the tumor microenvironment. These data indicate that HNK is a versatile anticancer agent that mediates linked pathways of oxidative stress, inflammation, cell cycle control, apoptosis, and metastatic signaling. These mechanistic data suggest a high potential for HNK to serve as a complementary/synergistic therapeutic candidate for the treatment of BrCa. The actions of HNK across BrCa therapeutic modalities are shown in Figure 1.

## 3. HNK with Chemotherapy: Synergistic Interactions

Preclinical studies suggest that HNK may enhance the responsiveness of tumor cells to certain standard BrCa therapies. In MDA-MB-231 cells, combining HNK with PTX leads to higher levels of apoptosis and greater disruption of microtubules than PTX alone, suggesting enhanced mitotic stress and cell death [9]. The same synergy has been evaluated in multidrug-resistant (MDR) cancer models, in which HNK increases the efficacy of PTX by inducing apoptosis and overcoming efflux-mediated resistance, which suggests a generalized chemo-sensitizing effect of HNK for taxane-based regimens [32]. HNK increases DOX levels in BrCa cells [15], activating ROS-mediated apoptosis and cytotoxicity [33]. This is consistent with the more general finding that HNK boosts oxidative stress and pro-apoptotic signaling in tumor cells and suppresses chemoresistance. Recent reviews show that the capacity of HNK to support cancer combination therapy is based on its regulation of apoptosis, autophagy, and drug transport [10,34]. Translational techniques leverage interactions that are more effective in cancer cells. In a BrCa model, HNK nanosuspension-loaded, thermosensitive hydrogels as a local delivery system and systemic PTX had synergistic anti-tumor efficacy, with better targeted drug exposure and tumor suppression than systemic PTX alone [35]. Recent studies indicate that encapsulating PTX and HNK in MUC1-targeted planetary ball-milled (PBM) nanoparticles enhances cytotoxicity by lowering the IC_50_ values for the TNBC cell lines HCC70 and MDA-MB-231. This approach has demonstrated enhanced effects, suggesting that bioavailability and selectivity can be enhanced through the use of this dual–drug nanocarrier method [13]. These strategies helped overcome the HNK solubility and bioavailability shortcomings and retain or even enhance its capacity to sensitize tumors to chemotherapy [36]. These data support a rationale for HNK-based chemotherapy regimens in which HNK is not only an additive cytotoxic agent but also an active drug-response modulator that modulates redox status, apoptosis, and microtubule dynamics to facilitate therapy.

## 4. HNK Synergy with Targeted and Endocrine Therapies

Endocrine therapy is crucial for managing ER-positive BrCa cells, but endocrine resistance limits its long-term effectiveness. The resistance arises when tumor cells bypass ER-mediated growth through dysregulated signaling pathways, diminishing the efficacy of treatments such as selective estrogen receptor modulators (SERMs) and aromatase inhibitors. Key pathways contributing to resistance include MAPK, PI3K/AKT, mTOR, and CDK4/6, as well as alterations in estrogen receptor (ER) function, such as mutations or ligand-independent activation [37]. Simultaneous targeting of these interconnected networks with ER-directed therapies is essential for overcoming resistance and enhancing clinical outcomes in ER-positive BrCa [37]. HNK demonstrates significant antiproliferative activity in both hormone-dependent and hormone-resistant BrCa cells. HNK also shows promise when combined with targeted and endocrine therapies, especially in cases of pathway hyperactivation or acquired resistance. HNK restores sensitivity to estrogen signaling in endocrine-resistant MCF-7 cells and reduces tumor growth. Its combination with metformin increases cell death in resistant tumors [12]. HNK functionally mimics and complements the effects of mTOR pathway inhibitors by activating the LKB1–AMPK axis, thereby suppressing mTORC1 signaling and downstream translational machinery. Mechanistically, HNK-induced AMPK activation is essential for inhibiting phosphorylation of key mTORC1 substrates, p70S6K (p-S6K) and 4E-BP1, thereby attenuating protein synthesis and oncogenic signaling in BrCa cells [38].

Transcriptomic and network studies show that HNK modulates genes involved in tamoxifen resistance [15], including those involved in cell cycle regulation, survival signaling, and growth factor pathways, suggesting a mechanistic approach to overcoming endocrine resistance [15,39]. These interactions make HNK a potential adjuvant for resistant luminal and HER2-driven malignancies. HNK modulates hormone response signaling and growth factor signaling (e.g., PI3K/AKT/mTOR) to prevent cancers from escaping endocrine or targeted therapy. Further, HNK could reduce conventional treatment doses, reduce toxicity, and delay resistance by targeting convergent nodes of survival [10]. Therefore, HNK is useful for cancers with complex resistance phenotypes, in which single-pathway blockade typically has little effect because oncogenic pathways interact.

## 5. HNK in Immunotherapy and Multimodal Interactions

In addition to cytotoxic and targeted therapies, combining HNK with immunotherapy shows promise for enhancing immune responses. Recent evidence highlights the critical role of the tumor microenvironment (TME) in regulating both immunomodulation and metastasis of BrCa [40,41]. Studies suggest that the anti-tumor effects of HNK extend beyond metabolic reprogramming and may also modulate tumor immune responses by regulating HIF-1α [42,43]. In BrCa, HIF-1α serves as a central mediator linking hypoxia-driven glycolysis to immune evasion by upregulating glycolytic genes such as GLUT1, HK2, and PDK1 to sustain the Warburg effect [5,44] while simultaneously promoting an immunosuppressive tumor microenvironment through impairment of effector T cell and NK cell function and activation of immune escape pathways [45]. HNK disrupts this axis by promoting UFL1/BRE1B-mediated ubiquitination and degradation of HIF-1α, thereby suppressing glycolysis and reducing metabolic support for tumor growth [5]. Tumor glycolysis and lactate accumulation are well established to suppress anti-tumor immunity and contribute to resistance to checkpoint inhibitors; thus, HNK-mediated attenuation of HIF-1α signaling may alleviate both metabolic and immunological barriers within the tumor microenvironment [5,46,47,48]. HIF-1α–dependent hypoxic signaling has been demonstrated to induce epigenetic suppression of immune effector genes and promote resistance to PD-1 blockade in TNBC models [45]. This finding highlights its function as a central mediator linking metabolic processes and immunosuppression. Thus, targeting HIF-1α with HNK may offer therapeutic potential not only as a metabolic inhibitor but also as an adjuvant that enhances immunotherapy efficacy by reprogramming tumor immunometabolism. Supporting this concept, silencing HIF-1α using siRNA significantly improves the efficacy of combined chemotherapy (PTX) and immunotherapy (imiquimod) in BrCa by remodeling the tumor microenvironment, reducing immunosuppression, and promoting anti-tumor immune responses in mouse models [49]. Consistent with this immunomodulatory role, HNK has also been shown in other cancer models to enhance CD4^+^ and CD8^+^ T cell infiltration *in vivo* and suppress tumor growth by downregulating tumor cell PD-L1 expression, thereby blocking the PD-1/PD-L1 immune checkpoint axis and restoring anti-tumor immunity [42]. Moreover, Mei et al. (2023) showed that HNK limits TNBC lung metastasis by reprogramming macrophages [14] in the 4T1 mouse model with a breast tumor. It inhibits IL-13-induced M2 polarization via STAT3/STAT6 suppression while promoting M1 polarization through STAT1, decreasing M2 markers (CD206, Arg1, and CCL2) and increasing M1 markers (CD11c, iNOS, and IL-12). This shift lowers the M2/M1 ratio and the IL-10/IL-12 balance, thereby reducing tumor invasion, migration, proliferation, and metastasis in murine BrCa. Preclinical findings further suggest that, in resistant or high-risk settings, rational combination strategies, potentially enabled by advanced delivery platforms such as nanoparticles and hydrogels, may offer meaningful clinical benefits. Therefore, HNK appears capable of simultaneously targeting tumor metabolism and immune evasion pathways, highlighting its potential for synergy with immune checkpoint blockade. Accordingly, HNK may serve as an adjuvant agent that could enhance the overall efficacy of BrCa therapy.

## 6. Honokiol and Drug-Resistance Mechanisms in BrCa

HNK reduces drug resistance in BrCa by acting on cancer stem cells, drug-efflux mechanisms, and survival pathways that allow resistant cells to persist. In SUM149, SUM159, and MDA-MB-231 cells [22], HNK lowers STAT3 activity [20] and reduces the expression of stemness markers such as Sox2 and Nanog, resulting in fewer mammospheres and diminished stem-like behavior [22]. In DOX-resistant MCF-7/ADR cells, HNK partially reverses MDR by promoting apoptosis, increasing intracellular drug retention, and decreasing P-glycoprotein (P-gp) expression [50,51]. Additionally, HNK increases the response to trastuzumab [52] and improves drug sensitivity in the HER2^+^ model, BT-474, by suppressing PI3K/AKT/mTOR [53] and STAT3 signaling [20]. Following HNK therapy, MCF-7/TAMR cells show restored ER signaling and lower proliferation, indicating a similar impact on endocrine resistance [12,54]. Thus, for ER^+^, HER2^+^, TNBC, and chemo-resistant BrCas, HNK inhibits resistance mechanisms.

Apart from these cell-intrinsic effects, HNK affects the tumor environment in ways that prevent resistance from developing. By inhibiting oxidative stress and inflammatory signaling, HNK minimizes conditions that support the survival of resistant clones and the preservation of stem-like populations [4]. Animal studies show that HNK improves treatment response by limiting tumor growth [29], supporting its potential use as an adjunct rather than as a replacement for existing therapies.

## 7. Preclinical Results for HNK in Treatment of BrCa

**Figure 2:**
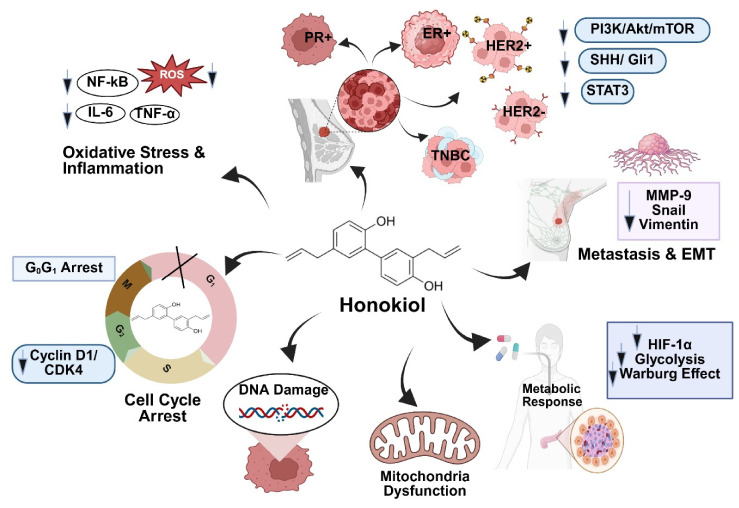
The multifunctional anticancer actions of honokiol (HNK) in BrCa. Honokiol targets various BrCa subtypes by addressing inflammation, cell cycle progression, metabolism, and metastatic signaling. It reduces NF-κB-driven inflammation and oxidative stress, induces G_0_/G_1_ arrest by suppressing cyclin D1–CDK4, and promotes DNA damage. It inhibits metastatic processes by downregulating MMP-9, Snail, and vimentin while modulating key pathways such as PI3K–Akt–mTOR, SHH–Gli1, and STAT3. HNK also suppresses HIF-1α-dependent glycolysis and the Warburg effect, demonstrating its role as a multitarget modulator in BrCa.

In BrCa, HNK functions as a metabolic and signaling modulator, affecting processes, including hypoxia [55], proliferation, apoptosis, and metastasis [21]. The regulation of hypoxia-adaptive processes by HIF leads to BrCa development and therapy resistance, making it a target for personalized, biomarker-based treatments [55]. HNK suppresses HIF-1α-controlled glycolysis by downregulating glycolytic metabolic enzymes, disrupting glucose uptake, and inhibiting tumor growth. It targets the Warburg effect and the metabolic plasticity of BrCa [5,33,56]. These findings are consistent with previous research indicating that HNK affects oncogene signaling pathways, including STAT3, EGFR/PI3K/AKT/mTOR [53], and Wnt/β-catenin [57]. This leads to elevated levels of cleaved PARP and lower expressions of Bcl-2 and ERα in hormone-resistant cancer cells. In due course, these effects overcome endocrine resistance and modify energy metabolism [12,34]. The multitarget anticancer mechanisms of HNK in BrCa are shown in Figure 2. In hormone-resistant BrCa cells, HNK and metformin work together to suppress cell proliferation and survival signaling, supporting a dual-targeting strategy against oncogenic pathways and tumor metabolism. These findings corroborate earlier research showing that HNK impairs mitochondrial integrity, enhances ROS-induced apoptosis, and sensitizes BrCa cells to both standard and targeted chemotherapeutics [5,12,16,58]. The mechanisms by which HNK interacts with other therapeutic agents to enhance the efficacy of therapy for BrCa cells are listed in Table 1.

Studies of drug delivery have led to elevated translational applicability of HNK [62]. In BrCa models, various nanotechnology formulations, including liposomes, polymeric micelles, solid lipid nanoparticles, and hybrid systems, have been examined to enhance the solubility, pharmacokinetics, and intratumoral delivery of HNK [63]. For xenograft and 4T1 models, HNK-loaded nanocarriers combined with standard chemotherapeutics such as PTX or DOX inhibit tumor growth, increase apoptotic indices, and reduce metastatic burden [11]. A separate study featuring mesoporous polydopamine nanoparticles loaded with HNK demonstrates that HNK, when combined with low-dose metformin and photothermal therapy, inhibits BrCa growth and increases apoptosis, confirming a role of HNK in nanomedicine platforms [61]. 

Lipid-based nanocarriers, such as liposomes and solid lipid nanoparticles (SLNs), are among the most clinically advanced drug delivery platforms. These carriers enhance HNK solubility and stability and facilitate passive tumor targeting via the enhanced permeability and retention (EPR) effect. In BrCa, lipid nanoparticles exhibit high biocompatibility, increased drug bioavailability, and reduced systemic toxicity relative to conventional formulations. Nevertheless, moderate drug-loading capacity and potential stability concerns during storage continue to present significant challenges [64,65].

Polymeric nanoparticles, particularly poly (lactide-co-glycolide) (PLGA) and PEG-based systems, provide controlled drug release and improved pharmacokinetics [66]. PLGA is a biocompatible, biodegradable polymer with high drug-loading capacity, enabling dual targeting via EPR and ligand-mediated mechanisms [67]. Haggag et al. (2020) showed that HNK-loaded PEGylated PLGA nanocapsules significantly enhanced cellular uptake, inhibited BrCa cell growth (80.2% vs. ~35% for free HNK), and prolonged circulation compared to the free drug [66]. Accordingly, PEGylated nanoconjugates were found to be safe and to significantly inhibit angiogenesis and enhance apoptosis in an *in vivo* model.

Hybrid and hydrogel-based systems improve HNK delivery by enabling localized, sustained release within the tumor microenvironment, thereby minimizing systemic exposure. HNK nanosuspensions incorporated into thermosensitive injectable hydrogels (HK-NS-Gel) demonstrated optimal gelation characteristics and enabled controlled HK release for up to 12 days. When combined with PTX, this approach achieved 72.51% tumor growth inhibition, indicating its potential as a strategy for BrCa therapy [35].

From a pharmacokinetic perspective, nanocarrier-based delivery consistently improves HNK performance by enhancing solubility, prolonging circulation time, and enabling targeted accumulation in tumors. These advantages are directly associated with improved therapeutic outcomes, including increased tumor suppression and reduced off-target toxicity. Furthermore, various nanocarriers offer distinct release profiles, which enable the optimization of treatment strategies according to disease stage and specific therapeutic objectives [62].

Toxicity profiles differ among nanocarrier platforms but are generally favorable for lipid-based and biodegradable polymeric systems. Liposomes and albumin-based nanocarriers have demonstrated clinical safety and regulatory acceptance, as evidenced by several FDA-approved formulations for cancer therapy [68]. Similarly, poly(lactic-co-glycolic acid) (PLGA) nanoparticles are widely recognized for their biocompatibility and low toxicity [67], although long-term accumulation and immune responses require further evaluation. Despite the promise of nanoparticle-based therapies, safety concerns persist due to unique biological interactions, including long-term biodistribution, potential toxicity, immune responses, oxidative stress, inflammation, and possible genotoxic effects [63,69]. These observations indicate that no single nanocarrier platform is universally optimal; rather, each offers distinct advantages depending on the therapeutic context. Lipid-based systems are notable for safety and clinical readiness; polymeric nanoparticles provide enhanced control over pharmacokinetics and drug release; and hydrogel-based systems facilitate localized, sustained delivery. Combining these platforms with HNK’s capacity to target tumor metabolism and immune evasion pathways further enhances its potential as a candidate for combination therapy in BrCa.

## 8. Clinical Evidence for the Use of HNK

Although preclinical evidence shows impressive anticancer properties of HNK, there are limited clinical trials of HNK that remain in an early stage, and no registered clinical trials have specifically studied HNK in the treatment of BrCa. The human data are primarily based on small case-level experiences, which, however, provide valuable preliminary information about safety, feasibility, and potential therapeutic relevance.

The strongest clinical evidence is provided by two patients who had progressed solid tumors that were drug-resistant and who had received intravenous HNK in an integrative oncology program [2]. Although neither case relates to BrCa, the report can be considered the first documented case of systemic administration of HNK in humans. To treat patients, intravenous doses were increased to 50 mg/kg, and the treatment was well tolerated with no dose-limiting toxicity. Both subjects showed improvement in quality of life, characterized by increased energy and appetite, and one patient demonstrated stabilization of radiographic conditions. The results provide data that establish the safety background and demonstrate that high-dose, systemic HNK can be achieved in the clinic, thereby justifying consideration of future early-phase clinical trials.

Another clinical report is for one patient undergoing oral HNK therapy for recurrent glioblastoma after being on standard therapy [70]. The primary finding in this instance was radiographic tumor regression and clinical improvement with adjunctive HNK administration, indicating possible biological activity even for a malignancy that is highly resistant to treatment. Although not related to BrCa, this finding suggests that HNK may exert anti-tumor effects in humans and warrants further investigation.

Moreover, a new early-phase clinical trial (NCT06566443) has commenced; however, it does not target BrCa. This Phase I study is assessing the safety and tolerability of HNK as a dietary supplement in individuals with early-stage, resectable non-small cell lung cancer. The trial aims to enroll approximately 15 participants and includes a short treatment period prior to surgery. The primary objective is to determine the maximum tolerated dose (MTD), rather than to evaluate therapeutic efficacy. This investigation constitutes an initial, safety-oriented study in humans, characterized by a narrow scope, a specific disease population, and a limited sample size. As a result, it yields only preliminary data on safety and tolerability and provides no evidence of anticancer activity or clinical benefit. Therefore, any discussion of its effects should be interpreted cautiously. Importantly, the current evidence supporting HNK activity in BrCa is derived almost exclusively from *in vitro* and animal studies.

Recent reviews have highlighted early-phase clinical trials assessing HNK in BrCa, although these trials remain unregistered. It is necessary to organize clinical development to convert strong preclinical results into human trials. Given HNK’s capacity to regulate oncogenic pathways, increase chemosensitivity, and orchestrate tumor metabolism and immune responses in preclinical BrCa models, early-phase clinical trials, especially dose-escalation trials of intravenous or nanoparticle-formulated HNK, are warranted. These studies could provide information on HNK pharmacokinetics, safety, and efficacy and may form the basis of studies that incorporate HNK with chemotherapy, endocrine therapy, targeted agents, or immunotherapy.

## 9. Challenges and Future Directions for HNK in BrCa

HNK exhibits favorable membrane permeability due to its lipophilic structure, enabling rapid, extensive tissue distribution and facilitating passage across the blood–brain barrier (BBB). Nevertheless, these physicochemical characteristics, particularly lipophilicity, contribute to poor aqueous solubility and limited oral bioavailability. Lipinski’s Rule of Five predicts that compounds with a log Pₒ/*w* greater than 5 will have suboptimal absorption and permeability. Both HNK and magnolol meet this criterion, reflecting their unfavorable oral pharmacokinetic profiles [71,72]. Despite the anticancer activity of HNK, its clinical translation in BrCa remains limited. This limitation is primarily attributed to pharmacokinetic challenges, including poor aqueous solubility, low oral bioavailability, rapid metabolism, and the lack of standardized dosing regimens, which together hinder consistent therapeutic exposure [73].

HNK modulates multiple signaling pathways, including PI3K/AKT/mTOR [74], EGFR [19], STAT3, NF-κB [60], and key metabolic networks, leading to context-dependent effects that vary across tumor subtypes and microenvironments. This lack of selectivity complicates the identification of predictive biomarkers, the development of optimal dosing strategies, and the reliable assessment of target engagement in clinical settings. While preclinical studies indicate a favorable toxicity profile, the potential for off-target effects in normal tissues remains insufficiently characterized in humans. Addressing these challenges will require future studies on targeted delivery strategies rather than a one-size-fits-all strategy approach.

Preclinical studies demonstrate that following oral administration at 40 mg/kg, HNK is rapidly absorbed (Tmax ≈ 20 min) but exhibits low systemic exposure [75], due to extensive first-pass metabolism and high hepatic extraction [76]. The pharmacokinetic profile is biphasic, characterized by rapid distribution, slower elimination, and significant formation of circulating conjugated metabolites. While the plasma elimination half-life is moderately prolonged (t½ ≈ 290 min), clearance from tissues such as the liver, kidney, and brain occurs more rapidly, suggesting compartment-specific pharmacokinetics [75].

The clearance of HNK is primarily mediated by Phase II metabolic processes, particularly glucuronidation and sulfation [76]. Consistent with this, oral administration leads to low and variable plasma concentrations, which complicates the maintenance of therapeutically relevant levels demonstrated in preclinical models [73]. Extensive hepatic metabolism produces a very high extraction ratio (E ≈ 0.99), moderate clearance (~35.8 mL/min), and extremely low bioavailability (F ≈ 0.007), indicating that conjugative pathways, mainly involving UDP–glucuronosyltransferases and sulfation enzymes, are the predominant mechanisms of drug elimination [76].

Similar pharmacokinetic limitations are observed with the related compound 4-O-methylhonokiol, including high systemic clearance, rapid metabolic degradation, and low bioavailability (Cmax 24.1 ± 3.3 ng/mL at 2.9 ± 1.9 h), which suggests that metabolism, rather than absorption, limits systemic exposure [77]. HNK interacts with cytochrome P450 (CYP) enzymes, acting as a weak inducer of CYP2B6 at high concentrations, but does not significantly affect other major CYP or Phase II enzymes, indicating a low probability of clinically relevant drug–drug interactions [78]. Additional studies report that HNK may compete for CYP3A4-mediated metabolism, thereby influencing the pharmacokinetics of co-administered drugs and functioning as both a substrate and modulator of drug-metabolizing enzymes [79]. Due to its interactions with key metabolic enzymes, including the CYP3A, CYP1A [79], and CYP2C families [80], HNK may alter enzyme activity and modify the systemic exposure of co-administered compounds, thereby presenting a potential risk of pharmacokinetic drug–drug interactions. These challenges underscore the necessity for optimized formulations, such as surfactant-based and nanocarrier systems, to enhance HNK exposure and consistency.

Nanomicellar and nanoparticle formulations have demonstrated improved HNK bioavailability, particularly in TNBC models, by increasing absorption, Cmax, and area under the curve (AUC), as well as enhancing tumor suppression in xenograft studies [36,81]. However, these approaches have not yet progressed to human clinical trials [81]. The inability to develop uniform formulation and dosing models across studies also complicates comparisons and limits the ability to establish exposure–response relationships needed to design clinical trials.

Another issue is the scarcity of clinical data supporting the use of HNK in BrCa patients. To date, there has been limited clinical evidence; most of the available evidence is based on *in vitro* research and animal models, making it challenging to determine the safety, efficacy, and pharmacokinetics in humans [2,4]. The differences between BrCa subtypes and experimental systems also make the results difficult to interpret and to identify biomarkers that may predict a more challenging therapeutic response. To enhance translational relevance, comprehensive pharmacokinetics and toxicology studies, optimization of the delivery system to increase systemic exposure, and extensive testing of HNK in combination with existing therapies are necessary. Lastly, Phase I and II clinical trials will be required to identify therapeutic windows and clinical benefit so that HNK can be evaluated as a single therapy or as adjuvant therapy for BrCa.

## 10. Conclusions

HNK has shown potential anticancer activity in breast cancer (BrCa) models. It interferes with metabolic and inflammatory networks that support tumor growth, suppresses proliferative and survival signals, enhances apoptosis, and reduces metastatic behavior in BrCa subtypes. Research from *in vitro* and animal studies indicates that HNK may boost the effectiveness of chemotherapy, endocrine therapy, targeted agents, and immunotherapy. This indicates its potential as an adjuvant to improving treatment responses and reducing drug resistance. Current studies suggest that HNK has little or no toxicity and may possess anti-tumor activity. However, these findings are largely derived from preclinical systems, and clinical evidence remains extremely limited, with no dedicated trials in BrCa and only isolated reports in other malignancies. Advances in nanoparticle-based delivery systems may help address challenges related to solubility and bioavailability, potentially improving translational feasibility. Nonetheless, the current evidence base should be considered exploratory and hypothesis-generating rather than definitive. Substantial gaps remain in understanding HNK’s pharmacokinetics, safety profile, optimal dosing, and therapeutic efficacy in humans. Further investigations should prioritize well-designed clinical studies, alongside efforts to standardize formulations, identify predictive biomarkers, and define rational combination strategies.

## Figures and Tables

**Table 1:** Mechanisms of action of HNK in synergy with other drug partners against BrCa cells.

Drug Partners	Model/Cell Types	Mechanism of Action	Outcome	References
Doxorubicin (DOX)	MCF-7, MDA-MB-231 cells	Suppress MRP1 (ABCC1); target, MUC1-MRP1 axis.	Enhanced chemosensitivity and improved therapeutic effectiveness; cell growth inhibition; enhancing anti-tumor activity.	[59]
Trastuzumab	HCC1954 cells	Regulate ER signaling; modulates PI3K/AKT, STAT3, and ERα (ESR1) signaling	Increased cytotoxicity in resistant cells (TR-HCC1954); restores trastuzumab sensitivity.	[52]
Rapamycin	BT-474 cells	Augments mTOR inhibition by suppressing PI3K/Akt/mTOR; suppresses S6K signaling.	Reduces adaptive survival signaling and cell proliferation and enhances growth inhibition and apoptosis.	[53,60]
Tamoxifen	MCF-7/TAMR cells	Restores ER sensitivity in tamoxifen-resistant cells by suppressing survival and cell-cycle pathways. resulting in reduced tumor growth.	Reversal of endocrine resistance and reduction in tumor growth and cell proliferation in tamoxifen-resistant models.	[15]
PD-1 blockade	4T1 murine model, and other cancers	Enhances immunotherapy efficacy by reducing inflammatory cytokines and boosting CD4^+^ and CD8^+^ T-cell activity.	Greater anti-tumor immunity and improved tumor regression.	[14,42]
Metformin	MCF7; MDA-MB-231, and SKBR3	Accumulation of cleaved PARP; downregulation of Bcl-2 and ERα; induces apoptosis.	Limits tumor growth.	[12,61]
Chloroquine	MCF7, MDA-MB-231, HCC1569, BT549, and MDA-MB-468	Activates autophagy markers: LC3B-II conversion, ATG protein expression, autophagosome formation, and autophagosome–lysosome fusion.	Inhibition of tumor growth and metastasis.	[34]
Nanocarrier systems	Xenograft models and the 4T1 model	Improves HNK bioavailability and enhances apoptosis, tumor suppression, and chemotherapy sensitization.	Improved drug delivery, tumor suppression, and reduced systemic toxicity.	[11]
PBM Nanoparticle	HCC70 and MDA-MB-468 cells	Induces cell death via suppression of MUC1.	Improved cytotoxicity compared to free drug, HNK	[13]
Paclitaxel with HNK dual drug-loaded PEOz-PLA micelles	MCF-7/ADR, and MDA-MB-231 cells	Enhanced cytotoxicity in drug-resistant BrCa cells; Induces anti-invasion and anti-migration in BrCa cells; P-gp inhibition; and MMP inhibition.	Increased chemosensitivity; Suppressed MDR and metastasis of BrCa	[9]

## Data Availability

This article includes all the data generated and analyzed in the study.
